# The Australian national binge drinking campaign: campaign recognition among young people at a music festival who report risky drinking

**DOI:** 10.1186/1471-2458-11-482

**Published:** 2011-06-20

**Authors:** Caroline van Gemert, Paul Dietze, Judy Gold, Rachel Sacks-Davis, Mark Stoové, Hassan Vally, Margaret Hellard

**Affiliations:** 1 Centre for Population Health, Burnet Institute, Melbourne, Australia; 2 National Centre for Epidemiology and Population Health, Australian National University, Canberra, Australia; 3 School of Public Health, La Trobe University, Melbourne, Australia

## Abstract

**Background:**

The Australian Government launched a mass media campaign in 2009 to raise awareness of the harms and costs associated risky drinking among young Australians. The aim of this study was to assess if young people attending a music festival who report frequent risky single occasions of drinking (RSOD) recognise the key message of the campaign, "*Binge drinking can lead to injuries and regrets*", compared to young people who report less frequent RSOD.

**Methods:**

A cross-sectional behavioural survey of young people (aged 16-29 years) attending a music festival in Melbourne, Australia, was conducted in January 2009. We collected basic demographics, information on alcohol and other drug use and sexual health and behaviour during the previous 12 months, and measured recognition of the Australian National Binge Drinking Campaign key message. We calculated the odds of recognition of the key slogan of the Australian National Binge Drinking Campaign among participants who reported frequent RSOD (defined as reported weekly or more frequent RSOD during the previous 12 months) compared to participants who reported less frequent RSOD.

**Results:**

Overall, three-quarters (74.7%) of 1072 participants included in this analysis recognised the campaign message. In the adjusted analysis, those reporting frequent RSOD had significantly lower odds of recognising the campaign message compared to those not reporting frequent RSOD (OR 0.7, 95% CI 0.5-0.9), whilst females had significantly greater odds of recognising the campaign message compared to males (OR 1.8, 95% CI 1.4-2.1).

**Conclusions:**

Whilst a high proportion of the target group recognised the campaign, our analysis suggests that participants that reported frequent RSOD - and thus the most important group to target - had statistically significantly lower odds of recognising the campaign message.

## Background

Risky single occasion drinking (RSOD), so-called 'binge' drinking, by young people is a significant public health problem in Australia. RSOD is associated with increased risk from injury including vehicular accidents, drowning, accidental injury, and violence, as well as an increase in wider risk-taking behaviour among young people, particularly in terms of unsafe sex choices, sexual coercion and drink driving [1-6]. There is a growing body of evidence to suggest that many young Australian's engage in frequent RSOD; for example, findings from the 2007 National Drug Strategy Household Survey showed that monthly or more frequent RSOD was reported among over one-quarter of teenagers aged 14-19 years and among over one-third of young people aged 20-29 years [7]. Other household and school student surveys have shown similar findings, although using slightly different measures of RSOD [5,7-9].

In response to this issue, the Australian Government Department of Health and Ageing launched a $53.5 million National Binge Drinking Strategy in 2009 to raise awareness of the harms and costs associated with RSOD among young Australians [10]. The principal component of the Strategy was a two-year $20 million harm minimisation and behaviour change mass media campaign. Launched in November 2008, the campaign, titled "*Don't Turn a Night Out Into a Nightmare*" (hereafter referred to as the Drinking Nightmare Campaign), targeted young people aged 15-25 years and their parents [10]. The Drinking Nightmare Campaign incorporated a range of mass media strategies and outlets that appeal to and are used by young people including television, cinema, radio, online advertising, brochures and out-of home print advertisements such as free postcard advertising, washroom mirrors in nightclubs, street posters, stencil chalking and on street furniture [11]. Vignettes using shared images across the different media conveyed four different scenarios demonstrating the consequences of RSOD. Campaign images presented scenes of young people drinking alcohol followed by a scene illustrating a serious negative consequence of intoxication for the same young people and a statistic on the harms and consequences of RSOD relevant to the scene [11]. The vignettes are described in detail elsewhere [11,12]. The media plan for implementation of media strategies is presented in Table 1. The range of media strategies were implemented concurrently in the months following campaign launch; additional information regarding the actual frequency and coverage of individual media strategies are not publicly available. The aim of the Drinking Nightmare Campaign was to raise awareness of the harms and costs associated with RSOD, and to deliver personally relevant messages to encourage, motivate and support the primary target groups to modify their behaviour [11].

**Table 1:** Media plan for the implementation of the National Binge Drinking Campaign

	November		December				January				February					March				April			
Television	x	x	x	x																			
	
Magazine/Print	x	x	x	x	x	x	x	x	x	x	x	x	x	x	x	x	x	x	x	x	x	x	x
	
Radio			x	x			x	x			x	x			x	x	x	x	x	x	x	x	x
	
Digital/Internet	x	x	x	x	x	x	x	x	x	x	x	x	x	x	x	x	x	x	x	x	x	x	x
	
Cinema	x	x	x	x	x	x	x	x	x	x													
	
Out-of-home^1 ^print and other strategies	x	x	x	x	x	x	x	x	x	x	x	x	x	x	x								

*Source: Ipos-Eureka Social Research Institute "Australian Government Department of Health and Ageing National Binge Drinking Campaign - Evaluation Survey April 2009*"[11]

^1 ^Out-of-home print and other strategies include free postcard advertising, washroom mirrors in nightclubs, street posters and stencil chalking and on street furniture)

In recent years several mass media campaigns have been implemented internationally to address a range of substance use issues among young people, such as smoking, alcohol consumption and illicit drug use [13-19]. Mass media campaigns as a health promotion strategy have the capacity to reach a large and broad cross-section of the population, and are relatively inexpensive compared to other health promotion strategies. However, the evidence for behaviour change post mass media campaign interventions is mixed [16-21]. Given the high level of investment associated with mass media campaigns, it is important that campaigns are evaluated to ensure that both the target group is being reached and that campaigns are effective in initiating and sustaining positive behaviour change.

The aim of this study was to assess whether the Drinking Nightmare Campaign reached young people, and, if so, whether it effectively targeted young people who report RSOD. We used prompted recognition of key campaign messages as a proxy for reach, and identified correlates, including frequency of RSOD, of recognition of the key message of the campaign - that "*Binge drinking can lead to injuries and regrets*" among a sample of young music festival goers in Melbourne, Australia.

## Methods

### Study design

A cross-sectional behavioural survey of young people was conducted in January 2009 at a music festival in Melbourne, Australia. Participants self-completed a behavioural survey that included measures of sexual knowledge and behaviour, and alcohol and other drug use during the previous 12 months in addition to specific questions regarding recognition of the Drinking Nightmare Campaign.

### Setting

The Big Day Out is an annual music festival held in several cities in Australia and New Zealand in late January. The festival features a diverse range of music including popular contemporary rock music and electronic music performed by mainstream international acts and local acts. The Melbourne festival draws approximately 40,000 to 50,000 fans annually, predominantly young people.

### Recruitment and Eligibility

Participants were recruited by trained researchers via convenience sampling strategies at a market stall on festival grounds, and were eligible to participate if they were aged between 16 and 29 years and had sufficient English language skills to complete a self-administered questionnaire. Since 2005, the Burnet Institute has conducted annual cross-sectional surveys of young people at the Melbourne Big Day Out music festival; recruitment strategies have been described in detail previously [3,4,22].

### Outcome measurements

Recognition of the Drinking Nightmare Campaign message was a binary measurement, determined by asking participants if they had seen or heard advertisements relating to alcohol use by young people, and if they could identify the main message of the campaign from a range of plausible health promotion messages including: "*Binge drinking can lead to injuries and regrets*", "*Alcohol can cause long term damage to your liver*", "*Driving while drunk can cause you to lose your license*", "*Supplying alcohol to under 18 year olds is illegal*" or an open-ended response in which the participant could supply an alternative answer. The first message reported here was the only one included in the campaign.

### Explanatory variables

Frequent RSOD was measured as a dichotomous variable based on a single question, "*In the past 12 months, how often did you have SIX or more drinks on one occasion?*". Frequent RSOD included participants who reported consuming six or more drinks on one drinking occasion on a weekly or more frequent basis. Infrequent RSOD included those who reported consuming six or more drinks on one drinking occasion on a monthly or less frequent basis during the previous 12 months but not including people who did not report any RSOD during the previous 12 months. Participants who did not report RSOD during the previous 12 months were not included in the measurement of frequent/infrequent RSOD.

The following potential confounders were also collected and included in unadjusted and adjusted models: gender, age group (16-19 years, 20-24 years, 25-29 years), highest level of education completed (did not finish high school, high school, technical/tertiary education), living in an urban or regional area (based on Australian postcodes of residence classified according to proximity to major cities using the Australian Standard Geographical Classification Remoteness Areas system) [23,24], living arrangements (alone, with parents and/or other family members, with partner and/or housemates, other), illicit drug use in the previous month, multiple sexual partners in the previous 12 months (defined as having two or more sexual partners within the previous 12 months) and inconsistent condom use (defined as not consistently using condoms with new and/or casual partners, and/or regular partners if multiple regular partners were reported within the previous 12 months) in the previous 12 months.

### Statistical analysis

All participants who answered the Drinking Nightmare Campaign message recognition question were included in this analysis. Data were entered into a Microsoft Access database and statistical analysis was conducted in Stata version 9.1.

Unadjusted logistic regression was used to identify statistically significant candidate predictors (p < 0.05) for inclusion in a final adjusted model. The final model used reverse stepwise selection procedures in which all statistically significant predictors of campaign recognition were included in the initial model and removed sequentially until only significant predictors (p < 0.05) remained. Goodness of fit for both models was assessed using the Hosmer & Lemeshow test to 0.05 significance.

### Ethics

This study received ethical approval from the Alfred Hospital Human Ethics Committee in December 2008.

## Results

### Participation

A total of 1333 participants completed the study questionnaire. The overall participation rate could not be calculated; due to the fast pace nature of recruitment and the large crowds at the Big Day Out it was not possible to accurately record the total number of participants approached and the proportion that completed the questionnaire. Overall, 182 of the 1333 questionnaires were excluded because they were incomplete, missing a consent form or if the participant was outside of the age criteria. Of the 1151 valid surveys, an additional 79 questionnaires were excluded as they had missing values for the Drinking Nightmare Campaign recognition question. A total of 1072 questionnaires were included in this analysis.

### Sample characteristics

Socio-demographic and behavioural characteristics of the study participants are presented in Table 2. Of the 1072 participants included in analysis, almost two thirds (62.6%) were female and more than half (55.4%) were aged 16-19 years. The majority of the sample (60.9%) lived in a major city, and over half (59.7%) lived with their parents and/or other family members.

**Table 2:** Socio-demographic and behavioural characteristics of study participants

	n*^1^* (N = 1072)	%
**Gender**		
Male	398	37.1
Female	671	62.6
**Age Group**		
16 - 19 years	594	55.4
20 - 24 years	360	33.6
25 - 29 years	118	11.0
**Highest level of education^2^**		
Didn't finish high school	49	4.6
High school	543	50.7
TAFE/Tertiary	469	43.8
**Place of residence^3^**		
Major city	653	60.9
Regional	360	33.6
**Living arrangements**		
Alone	39	3.6
With parents and/or other family members	640	59.7
With partner and/or housemates	308	28.7
Other	50	4.7
**Reported alcohol and drug use behaviour**		
Reported RSOD at least once in the previous 12 months	928	86.6
Reported frequent RSOD*^4^*	316	29.5
Reported using illicit drugs in the past month	287	26.8
**Reported sexual health behaviours**		
Reported multiple sexual partners in previous 12 months*^5^*	389	36.3
Reported inconsistent condom use in previous 12 months^6^	292	27.2
**Exposure and recognition of campaign**		
Recognised campaign message	801	74.7

^1^Numbers of participants do not always add up to the total because of missing information for some variables

^2^Highest level of education includes those currently enrolled the specified level and those whose highest level is the specified level of education

^3^Place of residence was derived from postcode of residence using the Australian Standard Geographical Classification Remoteness Areas and only includes participants who provided an Australian postcode of residence

^4^Frequent RSOD was defined as participants who reported consumption of six or more drinks on one drinking occasion on a weekly or more frequent basis in the 12 months prior to the survey

^5^Multiple sexual partners was defined as having two or more sexual partners within the previous 12 months

^6^Consistency of condom use was defined as not consistently using condoms with new and/or casual partners, and/or regular partners if multiple regular partners were reported within the previous 12 months

Over one-third of the sample (36.3%) reported having multiple sexual partners in the previous 12 months and over one-quarter of all participants (27.2%) reported inconsistent condom use with sexual partners.

Of the 1072 participants, 928 (86.6%) reported RSOD at least once during the previous 12 months, of which just under one third (n = 316, 29.5%) reported frequent RSOD during the previous 12 months. Three-quarters (74.7%) of all participants recognised the message of the Drinking Nightmare Campaign.

### Correlates for recognition of campaign message

Unadjusted and adjusted odds ratios (OR/AOR) correlated with campaign recognition are presented in Table 3. Higher levels of campaign recognition were found among participants who reported infrequent RSOD compared to participants that reported frequent RSOD (79.2% vs. 68.7% respectively). In unadjusted analysis, reports of frequent RSOD in the previous 12 months were correlated with lower recognition of the Drinking Nightmare Campaign. The socio-demographic variables correlated with greater recognition of the campaign were females, participants whose highest level of education was technical or tertiary education, and participants who lived with their parents and/or other family members or with partner and/or housemates, whilst the socio-demographic variables correlated with lower recognition of the campaign were participants who reported multiple partners in the previous 12 months and inconsistent condom use. In the adjusted analysis, those reporting frequent RSOD had significantly lower odds of recognising the campaign message than those not reporting frequent RSOD (OR 0.7, 95% CI 0.5-0.9), whilst females had significantly greater odds of recognising the campaign message than males (OR 1.8, 95% CI 1.4-2.1).

**Table 3:** Unadjusted and adjusted odds ratios (OR/AOR) correlated with campaign recognition

	Recognised campaign message		Unadjusted analysis			Adjusted Analysis*^6^*		
	Total	Yes	Unadjusted OR	95% CI	p-value	Adjusted OR	95% CI	p-value
	n	%						
**Gender**								
Male	398	67.6	1.0					
Female	671	79.3	1.8	**1.4-2.4**	**< 0.01**	**1.8**	**1.4-2.5**	**< 0.01**
16 - 19 years	594	72.4	1.0					
**Age Group**								
20 - 24 years	360	76.9	1.3	0.9-1.7	0.10			
25 - 29 years	118	79.7	1.5	0.9-2.4	0.10			
**Highest level of education^1^**								
Didn't finish high school	49	63.3	1.0					
Finished/currently at high school	543	72.2	1.5	0.8-2.8	0.19			
Finished/currently at TAFE/Tertiary	469	78.9	2.2	1.2-4.0	0.12			
**Place of residence^2^**								
Major city	653	75.8	1.0					
Regional	360	73.1	0.9	0.6-1.2	0.34			
**Living arrangements**								
Alone	39	56.4	1.0					
With parents and/or other family members	640	74.2	2.2	**1.2-4.3**	**0.02**			
With partner and/or housemates	308	77.6	2.7	**1.3-5.3**	**0.01**			
Other	50	76.0	2.4	**1.0-6.1**	**0.05**			
**Reported frequent RSOD in previous 12 months^3^**								
No	612	79.2	1.0					
Yes	316	68.7	0.6	**0.4-0.8**	**< 0.01**	**0.7**	**0.5-0.9**	**0.01**
**Used illicit drugs in the previous month**								
No	761	76.0	1.0					
Yes	287	71.8	0.8	0.6-1.1	0.17			
**Reported multiple sexual partners in previous 12 months^4^**								
No	440	77.5	1.0					
Yes	389	70.2	0.7	**0.5-0.9**	**0.02**			
**Reported inconsistent condom use in previous 12 months^5^**								
No	410	79.5	1.0					
Yes	292	71.2	0.6	**0.5-0.9**	**0.01**			

^1^Highest level of education includes those currently enrolled the specified level and those whose highest level is the specified level of education

^2^Place of residence was derived from postcode of residence using the Australian Standard Geographical Classification Remoteness Areas and only includes participants who provided an Australian postcode of residence

^3^Frequent RSOD was defined as participants who reported consumption of six or more drinks on one drinking occasion on a weekly or more frequent basis in the 12 months prior to the survey

^4^Multiple sexual partners was defined as having two or more sexual partners within the previous 12 months

^5^Consistency of condom use was defined as not consistently using condoms with new and/or casual partners, and/or regular partners if multiple regular partners were reported within the previous 12 months

^6^Hosmer-Lemeshow: p > 0.05

## Discussion

This study presents the first independent process evaluation assessing recognition of key messages of a national harm minimisation and behavioural change campaign to address RSOD by young people in Australia and internationally. We found that whilst the campaign was recognised by the majority of our sample, those who constitute the greatest risk group for injury, death and other measures of short term harm - participants who report frequent RSOD [1-6] - had lower odds of recognising the key message of the Drinking Nightmare Campaign than participants who reported less frequent RSOD. This has important public health implications, particularly in terms of the development of health promotion campaigns that aim to target young people.

Just under one third of young music festival goers in this study reported frequent RSOD. This is slightly higher than estimates presented in other surveys; for example, 20% of participants in the Victorian Youth Alcohol and Drug Survey (VYADS) reported weekly RSOD [9], whilst 23.2% of young people aged 14-19 and 24.7% of young people aged 20-29 years participating in the National Drug Strategy Household Survey (NDSHS) and whom reported RSOD on at least one occasion in the previous 12 months reported frequent RSOD (defined as drinking seven or more drinks on one day for males, and five or more drinks on one day for females on a weekly basis) [7]. The difference in reported RSOD levels may be due to the recruitment of young festival goers rather than young people generally. Nonetheless, the ability of this study to recruit and distinguish between participants that report different alcohol consumption patterns is important and has not been reported elsewhere. This data provides an opportunity to assess the proportion of a risky sub-population of young people the Drinking Nightmare Campaign reached, and what pattern of alcohol consumption was correlated with recognition. Such analysis enables health promotion practitioners an opportunity to refine strategies to increase the likelihood that the most at-risk group of young drinkers - those that report frequent RSOD - are reached in future mass media campaigns.

Few mass media campaigns that aim to modify substance use by young people have been rigorously evaluated [25]. Among the published evaluations of mass media campaigns addressing substance use issues among young people reviewed here, the majority have utilised an impact evaluation framework and measured behaviour change in the short-term, post-implementation; the findings of these evaluations have been inconsistent [16-20]. Before the impact of a campaign can be measured, however, it is important that the implementation of a campaign is thoroughly evaluated. Mass media campaigns are based on the assumption that behaviour change is influenced by exposure to the campaigns and subsequent increased awareness and recognition of health promotion messages. It is therefore paramount to measure the extent to which the target audience was reached and awareness and recognition of campaign messages was achieved. Further, by identifying who is exposed to campaign messages, process evaluation provides important insights into the reach of mass media campaigns and how this affects the campaign objectives [26]. Despite the importance of process evaluations for mass media campaigns, we found few published evaluations that assess whether mass media campaigns addressing substance use among young people are successful in actually reaching their target audience and raise awareness of campaign messages [13,15]. The findings of these evaluations showed increased recall of messages after exposure to a mass media campaign [13] and that use of audience segmentation strategies (separate targeting of subgroups with common interests and message preferences rather than a single set of messages for the entire youth audience) may be an effective method to appeal to and resonate with diverse youth audiences [15]. These evaluations, however, did not go on to examine the effect of recall and awareness on moderating behaviour change.

With this in mind, our findings raise questions as to whether mass media campaigns are indeed the most appropriate strategy to reach young people who report frequent RSOD. The failure to reach this group may be governed by a variety of explanations related to exposure to mainstream media (for example, high sensation seekers may be less likely to be exposed to television or other media outlets used). Alternative social marketing strategies should be explored to understand how to best reach this at-risk group. For example, peer-based interventions, or the innovative use of new technologies such as mobile telephone interventions or social networking websites may be a more effective strategy to reach young people who report frequent RSOD [27-30], as may use of audience segmentation strategies to specifically target youth who engage in higher risk activities [31,32].

Overall, approximately three-quarters (74.7%) of all participants in our study recognised the key message of the Drinking Nightmare Campaign mass media strategy. This level of recognition is lower than what was reported in an evaluation of the campaign prepared for the Australian Government Department of Health and Ageing, which reported net campaign reach between 84-86% for 15-25 year olds [11]. This discrepancy may be due to a number of methodological differences such as measurement or timing. For example, the measure of campaign recognition used in the official evaluation was not clearly outlined and may differ from our measurement [11]. Further, our study was conducted two months after the campaign launch, while the official evaluation was conducted six months after the campaign launch and potentially following sustained and repeated exposure to the campaign during this time (as indicated in Table 1) [11]. Finally, this difference may be due to population differences in the evaluation sample compared to our study. Nonetheless, the official evaluation of the Drinking Nightmare Campaign did not explore campaign reach according to patterns of alcohol consumption and thus did not assess whether the campaign reached young people who reported frequent RSOD. Whilst data presented here and in the official evaluation of the Drinking Nightmare Campaign suggest that a high proportion of the target group recognised the campaign, our analysis suggests that those most at-risk - and thus the most important to target - had significantly lower odds of recognising the campaign.

This analysis has some limitations. Firstly, participants were recruited in a convenience sample at a music festival and may not be representative of young people in general; however, this recruitment strategy is appropriate given that it was effective in recruiting the group of interest; that is, young people who report RSOD. Secondly, we were unable to calculate the overall participation rate and thus we are unable to determine if there was a difference between participants and non-participants. We note that researchers actively sought participants by inviting all people passing the market stall to participate, rather than self-selection of participation, which is likely to reduce selection bias; further, researchers worked at full capacity throughout the day suggesting a high rate of participation. Thirdly, there is the potential that participants identified the correct message by chance through the use of aided recognition; participants were asked to identify the main message of the advertisements they had seen from a group of five plausible health promotion messages. The use of additional 'decoy' health promotion messages is likely to have improved the reliability of this response however it is still possible that some participants nominated the correct message by chance without correctly recognising the campaign message. Finally, the study was conducted only two months after the launch of the campaign and some participants may have not had the opportunity to be exposed to the campaign. Whilst the reported level of campaign recognition was lower than what was reported in the evaluation prepared for the Australian Government Department of Health and Ageing, overall campaign recognition reported by participants in this study was nonetheless high and our analysis was able to detect a statistically significant difference between levels of recognition of key campaign messages between young people that reported frequent and infrequent RSOD.

## Conclusions

Whilst mass media strategies may be effective at reaching a broad cross-section of society, they may not reach the intended target group and may miss important subsections of the population. In this study, we have demonstrated that despite high levels of campaign recognition reported by participants, there was a difference between levels of recognition of key campaign messages between young music festival goers that reported less frequent and frequent RSOD. RSOD is a significant public health problem in Australia, and it is imperative that health promotion strategies reach those who report high-risk behaviours. Recognising this, these results show that health promotion campaigns need to develop more targeted and population-relevant strategies to reach those most vulnerable to the risks associated with frequent RSOD. We argue that future mass media strategies employed by the National Binge Drinking Campaign and similar campaigns addressing substance use by young people be redressed to ensure that they are evidence-based and effectively target at-risk groups of young people.

## List of abbreviations

NDSHS: National Drug Strategy Household Survey; OR/AOR: Unadjusted and adjusted odds ratios; RSOD: risky single occasion of drinking; VYADS: Victorian Youth Alcohol and Drug Survey

## Competing interests

The authors declare that they have no competing interests.

## Authors' contributions

CVG conducted the data analysis and prepared the manuscript. PD contributed to data analysis and interpretation of findings. MH and JG conceived of the study. JG, RSD and MH contributed to the study design and data collection. All authors contributed to and have approved the final manuscript.

## Pre-publication history

The pre-publication history for this paper can be accessed here:

http://www.biomedcentral.com/1471-2458/11/482/prepub
